# Lipid metabolic vulnerabilities of multiple myeloma

**DOI:** 10.1007/s10238-023-01174-2

**Published:** 2023-08-28

**Authors:** Roberta Torcasio, Maria Eugenia Gallo Cantafio, Raissa Kaori Ikeda, Ludovica Ganino, Giuseppe Viglietto, Nicola Amodio

**Affiliations:** 1 Department of Experimental and Clinical Medicine, Magna Graecia University of Catanzaro, Viale Europa, Campus Germaneto, 88100 Catanzaro, Italy; 2 Department of Biology, Ecology and Heart Sciences, University of Calabria, Arcavacata Di Rende, Cosenza, Italy; 3 Centro Universitário São Camilo, São Paulo, Brazil

**Keywords:** Cholesterol, Fatty acids, Lipid metabolism, Metabolic reprogramming, Multiple myeloma, Sphingolipids

## Abstract

Multiple myeloma (MM) is the second most common hematological malignancy worldwide, characterized by abnormal proliferation of malignant plasma cells within a tumor-permissive bone marrow microenvironment. Metabolic dysfunctions are emerging as key determinants in the pathobiology of MM. In this review, we highlight the metabolic features of MM, showing how alterations in various lipid pathways, mainly involving fatty acids, cholesterol and sphingolipids, affect the growth, survival and drug responsiveness of MM cells, as well as their cross-talk with other cellular components of the tumor microenvironment. These findings will provide a new path to understanding the mechanisms underlying how lipid vulnerabilities may arise and affect the phenotype of malignant plasma cells, highlighting novel druggable pathways with a significant impact on the management of MM.

## Introduction

Multiple myeloma (MM) is a biologically and clinically heterogeneous malignancy of terminally differentiated plasma cells (PCs), which abnormally proliferate in the bone marrow (BM), and typically secrete non-functional monoclonal immunoglobulin (Ig) in serum and urine. The stages of MM development usually begin from an asymptomatic premalignant condition defined monoclonal gammopathy of undetermined significance (MGUS), which can progress, through smoldering myeloma (SMM), to a clinically active disease and finally to plasma cell leukemia, where malignant PCs are no longer dependent from the BM for growth and survival. The standard clinical practice is to monitor MGUS and SMM patients, in absence of therapy, for their risk of progression to overt MM, which accounts to 1% per year for MGUS patients, and 10% per year for SMM patients within 5 years, declining to 3% per year thereafter [1, 2].

Despite notable advancements in treatment options targeting PCs within their tumor-promoting BM *milieu* (BMM), most of MM patients relapse and unfortunately succumb to the disease [1, 3], thus prompting the continuous search of new actionable vulnerabilities.

Cellular metabolism is the result of finely orchestrated fundamental processes which produce the energy necessary for maintaining cellular homeostasis thus supporting growth, proliferation, and differentiation [4]. During tumorigenesis, the accumulation of mutations in oncogenes and tumor suppressor genes may frequently account for the dysregulation of metabolic pathways [5, 6]. On this basis, dysfunctional metabolism has emerged as a key determinant in tumor pathobiology, providing new targets for therapeutic intervention [7–10].

Overall, cancer cells experience metabolic reprogramming to enhance energy production, which is mandatory to sustain their elevated biosynthetic needs and promote disease progression [11, 12]. Aerobic glycolysis, i.e., the process whereby cancer cells metabolize glucose even in the presence of oxygen, has been widely reported among cancer hallmarks, leading to important metabolic consequences, such as elevated ATP synthesis, increase of pentose phosphate pathway (PPP), and decrease in ROS and oxidative stress through generation of nucleotide precursors, producing acidity within the tumor microenvironment to sustain growth and metastasis [13]. Alongside, it has been recently demonstrated that cancer cells, by modulating their mitochondrial dynamics, can take advantage of high oxidative phosphorylation (OXPHOS), inhibiting the intrinsic apoptosis and leading to the resistance of cancer cells to apoptotic stimuli [6, 14–16].

Mitochondrial alterations, with increased biomass as well as unbalanced mitochondrial functions, can prompt metabolic changes which have been found instrumental in MM onset and chemoresistance [6, 16, 17]. MM cells are strictly dependent on glucose metabolism by enhancing glucose uptake and aerobic glycolysis and producing lactate, providing an important carbon source for tricarboxylic acid (TCA) cycle and OXPHOS, required to maintain elevated protein synthesis, folding and secretion. Accordingly, higher levels of lactate dehydrogenase (LDH) are associated with aggressive disease and poor prognosis of MM patients [18]. The enhancement of aerobic glycolysis also turns on the PPP to boost the generation of the antioxidant compounds, such as NADPH and glutathione (GSH), to reduce MM cells susceptibility to oxidative damage, making them more resistant to oxidative stress-inducing drugs [19]. Therefore, the inhibition of the glycolytic process or glycolysis-related biosynthetic pathways could reduce MM progression; accordingly, selective inhibition of GLUT1, found highly expressed and associated with worse outcomes of MM patients, antagonized glucose uptake eliciting anti-tumor activity in malignant PCs [20].

In addition to glucose, MM PCs seem highly dependent on amino acid metabolism for survival, exhibiting elevated glutamine uptake to fuel OXPHOS in the BM [21]. Accordingly, the oncogene c-MYC, widely overexpressed in MM, plays a key role in the regulation of aerobic glycolysis, by increasing glucose dependency as well as glutamine consumption through an increase in glutamine transporter (ASCT2) and glutaminase (GLS) transcription [22–24]. Indeed, alteration of glutamine uptake through inhibition of glutamine importer ASCT2 markedly reduced MM cell growth. Glutamine also plays a key role in proteasome inhibitors (PIs) resistance [10], and it is indispensable for the production of amino acids and nucleotides as well as for the synthesis of substrates for TCA cycle [25, 26]. Similarly, the elevated PPP and serine synthesis pathway sustain bortezomib resistance due to the increased anti-oxidant capacity in MM cells. Targeting serine metabolism can therefore enhance the sensitivity to bortezomib through inhibition of 3-phosphoglycerate dehydrogenase (PHGDH), which catalyzes the rate-limiting step for serine synthesis [27].

Importantly, metabolic reprogramming of MM cells is widely affected by bone marrow stromal cells (BMSCs), which surround and protect malignant PCs producing growth factors and cytokines as pro-survival signals [28]. Under the microenvironment hypoxic conditions, the mTOR (mechanistic target of rapamycin)-driven translation of the transcription factor HIF-1*α* (hypoxia inducible factor-1-*α*) is upregulated in MM, switching metabolism toward glycolysis through the overexpression of metabolic enzymes such as GLUT1, HK2 (hexokinase 2), LDH [29], and the pyruvate dehydrogenase kinase-1 (PDK-1) [30]; in turn, MM cells induce BMSCs to undergo aerobic glycolysis, producing metabolites for their utilization by neoplastic cells, as OXPHOS substrates, sustaining ATP production, and increasing cell fitness to foster growth and migration [31–34].

In the context of MM cell metabolism, the glucose pathway has been so far the subject of deep investigation, although more recent evidence highlights lipid vulnerabilities in MM PCs, providing new metabolic hubs to be targeted in this still largely fatal malignancy [35].

In this review, we summarize: (*i*) the major classes of lipids whose aberrant metabolism have been implicated in MM onset and chemoresistance, (*ii*) their impact on the activity of various BMM cell populations, and (*iii*) the potential strategies targeting lipid-related dependencies in preclinical models of PC dyscrasias. Finally, the potential of circulating lipids as biomarkers for MM patient therapy response prediction or risk stratification will be discussed.

## Lipid metabolic vulnerabilities of MM

Lipids are chemically defined as a diverse group of molecules, insoluble in water, not only components of organelles or cellular energy suppliers, but also signaling molecules crucial for maintaining cellular homeostasis [36].

Consistent with the seminal discovery in 1953 that tumors could synthesize lipids in a manner similar to embryonic tissues, many tumor cells have been found to exhibit a high rate of de novo lipogenesis [37]. Importantly, clinical observations found that lipid metabolism reprogramming often predicts poorer prognosis in cancer patients [38], including those affected by hematological malignancies. A large number of lipid droplets that store lipids and cholesterol can be detected in tumor cells, and high lipid droplets and cholesterol esters are nowadays considered indicators of aggressiveness of both solid and hematological cancers [38, 39].

**Fig. 1:**
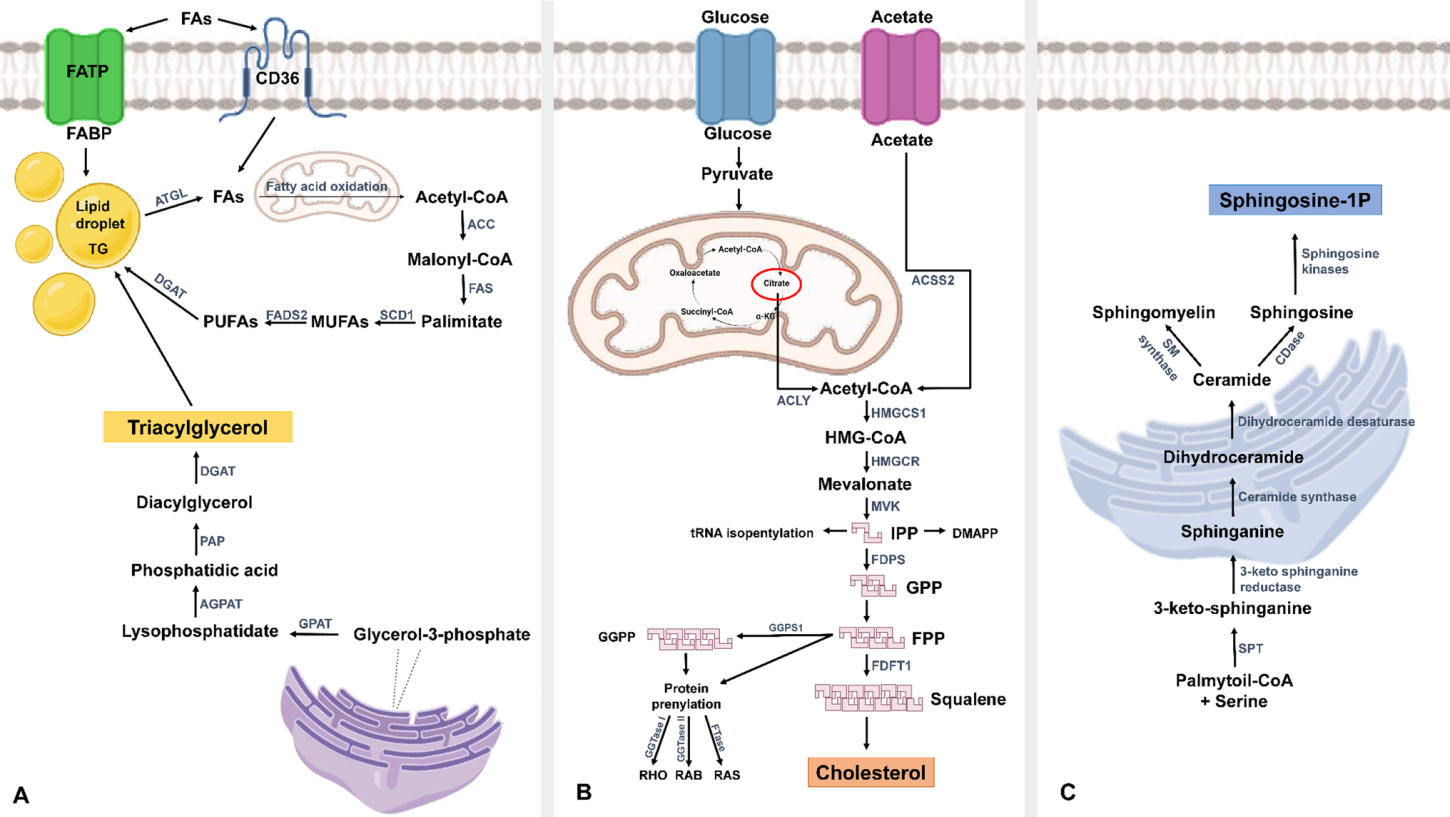
Graphic overview of the main lipid biosynthetic pathways found deregulated in MM. **A** FATP (Fatty acid transport protein) and CD36 (cluster of differentiation 36) proteins facilitate the transport of FAs through the plasma membrane to the cell compartments, where the synthesis of FAs takes place. Acetyl-CoA, derived from various metabolic sources, including glucose, amino acids, and mitochondrial FA oxidation, is carboxylated by the enzyme acetylCoA carboxylase (ACC) in the cytoplasm, requiring ATP and biotin as a cofactor. The carboxylation of acetyl-CoA results in the formation of malonyl-CoA, a building block for FA synthesis. The synthesis of FAs is instead carried out by a multi-enzyme complex called fatty acid synthase (FAS). Once the desired FA length is reached, the final product is typically palmitic acid (16 carbons). Palmitate can be desaturated by the enzyme stearoyl-CoA desaturase (SCD), which introduces a double bond between carbons 9 and 10 of the palmitate, resulting in the formation of MUFAs. PUFAs are synthesized from MUFAs by fatty acid desaturase 2 (FADS2). The synthesis of TGs in cells involves a series of steps within specific cellular compartments. Glycerol-3-phosphate, derived from glucose metabolism or glycerol uptake, serves as the starting point and it is converted to lysophosphatidic acid (LPA) by glycerol-3-phosphate acyltransferase (GPAT) in the endoplasmic reticulum (ER). LPA undergoes acylation by acyl-CoA to form phosphatidic acid (PA) in a reaction catalyzed by the enzyme acylglycerol-3-phosphate acyltransferase (AGPAT). The final step in the glycerol phosphate pathway involves the dephosphorylation of PA by phosphatidate phosphatase (PAP) to produce diacylglycerol (DAG). DAG is subsequently acylated by diacylglycerol acyltransferase (DGAT) to form triglycerides. Once synthesized, TGs are packaged into specialized cytoplasmic structures called lipid droplets. **B** Glucose transporters (GLUT) facilitate the passage of glucose through the cell membrane; glucose is then converted to pyruvate through glycolysis, and then enters the Krebs cycle as acetyl-CoA. Acetate also plays a key role as a precursor for cholesterol synthesis. Through a series of enzymatic reactions, acetate is activated with acetyl-CoA, which represents an important starting point for the synthesis of cholesterol. Then Acetyl-CoA, derived from various sources, including glucose metabolism, FA oxidation, and amino acid breakdown serves as a precursor for the synthesis of mevalonate, the initial step in cholesterol biosynthesis. AcetoacetylCoA then condenses with another molecule of acetyl-CoA, catalyzed by the enzyme HMG-CoA synthase, to produce 3-hydroxy-3-methylglutaryl-CoA (HMG-CoA), next converted to mevalonate by HMGCoA reductase; mevalonate is phosphorylated with the help of the mevalonate kinase (MVK) enzyme, and then undergoes a series of reactions forming isopentenyl pyrophosphate (IPP) and dimethylamil pyrophosphate (DMAPP), critical building blocks for the generation of various isoprenoids. Multiple IPP molecules can condense together to form longer isoprenoid chains, such as GPP (Geranyl Pyrophosphate), farnesyl pyrophosphate (FPP), and geranylgeranyl pyrophosphate (GGPP). FPP undergoes a series of reactions that lead to the formation of squalene, a precursor molecule of cholesterol; squalene is converted into cholesterol through a series of enzymatic reactions, which include the formation of lanosterol and the elimination of functional groups. In addition, geranylgeranyl diphosphate synthase 1 (GGPS1) converts FPP into geranylgeranyl pyrophosphate (GGPP). Prenylation, a process carried out by farnesyltransferase (FTase) and geranylgeranyltransferase (GGTase), allows the post-translational addition of both FPP and GGPP to various proteins, including those belonging to the RHO, RAS, and RAB families. **C** The synthesis of sphingolipids occurs primarily in the ER and starts with palmitoyl-CoA, a basic building block derived from the breakdown of FAs, that undergoes a series of enzymatic reactions, including condensation with serine, to produce 3-ketosphinganine. The 3-ketosphinganine is then reduced by the 3-ketosphinganine reductase, resulting in the formation of sphinganine that is acylated by ceramide synthase. This reaction leads to the formation of ceramide, which is the central molecule in sphingolipid synthesis. Different enzymes such as SM synthase and ceramidase (CDase) introduce modifications to ceramide to generate different types of sphingolipids, such as sphingomyelin and sphingosine. The final conversion of sphingosine to sphingosine-1-phosphate is catalyzed by sphingosine kinases. The picture was created using BioRender software

A graphic overview of the main lipid metabolic pathways found deregulated in MM, and discussed in the following sections of this review, is provided in Fig. 1.

### Fatty acids (FAs) and triglycerides

FAs play a key role in many cellular processes, contributing to the maintenance of the plasma membrane structural integrity as well as regulating cellular energy metabolism and signal transduction, ultimately impacting on cellular homeostasis [40]. Indeed, an aberrant FA metabolism has been linked to the cancer onset and progression, providing metabolites for cellular energy needs to support uncontrolled cell growth and proliferation [41]. Lipid supply can be accomplished either exogenously or, for certain lipid classes, by implementing biosynthetic pathways [42].

The synthesis of FAs is an anabolic process which occurs when the cellular energy need is diminishing, resulting in a reduction in TCA cycle pathway. FAs are taken up into the cells by using fatty acid translocase protein CD36 and by FA transporter proteins (FATP), whereas FA binding proteins (FABPs) shuttle FAs into the cells. Notably, FAs transported by CD36 are converted into secondary metabolites such as ceramides, diacylglycols and phospholipid inositol derivatives [35, 43–45]. FABPs facilitate the intracellular transport of long-chain FAs (LCFAs), and regulate lipid synthesis and oxidation [46]. In several tumors, an increased expression of CD36 has been found, which seems to promote the EMT process and thus a more aggressive tumor phenotype [47].

Once into the cells, FAs are activated by the addition of coenzyme A (CoA) mediated by the long-chain acyl-CoA synthetase (ACSL) group of proteins; at this point, they can undergo the esterification process in the ER or be re-routed to the mitochondria, through the carnitine palmitoyltransferase 1 (CPT1), where they are oxidized to produce Acetyl-CoA to support TCA cycle and further yield ATP [48]. The ACSL family plays an important role in cancer biology, and increased expression of ACSL has been observed in many types of cancer, such as colon, liver, lung, brain and colorectal cancers [46, 49–51].

According to the number of saturations and metabolic origin, it is possible to make a macrodistinction between saturated (SFA) and monounsaturated (MUFA) FAs, which originate from acetyl-CoA in cells, and polyunsaturated fatty acids (PUFA), synthesized from linoleic acid (C18:2n-6) and α-linoleic acid (ALA; C18:3n-3), two essential FAs introduced in the diet [42].

MUFA synthesis originates from the de novo lipogenesis. Starting from glucose which derives from dietary carbohydrates, after glycolysis and the TCA cycle, citrate is produced in the mitochondria, which is transported into the cytosol where is then released acetyl-CoA following the action of ATP-citrate lyase (ACLY). The resulting acetyl-CoA is converted to malonyl-CoA by acetyl-CoA carboxylase 1 (ACC1), and finally fatty acid synthase (FASN) serially condensates seven malonyl-CoA molecules to one acetyl-CoA to produce 16-carbon palmitate, which will undergo elongation (by ELOVL elongase enzymes) and desaturation (by FADS and SCD desaturase enzymes) reactions to generate bioactive FAs with different length and degrees of saturation, including stearic, palmitoleic and oleic acids [52]. ACC1 is the rate-limiting enzyme of de novo lipogenesis [53]; FAS enzyme is essential to produce bioactive lipids to sustain membrane structure and intracellular signaling. Since most cancers depend on the synthesis of new FAs, ACC1 and FAS represent potential therapeutic target for this disease [54].

During the genesis of PUFAs, the activity of some FA desaturases and elongases is essential for controlling the length and degree of unsaturation of FAs, thus influencing their functions and metabolic fate. One of the main enzymes with desaturase activity is the stearoyl-CoA desaturase-1 (SCD1), localized at the ER, catalyzing the biosynthesis of MUFAs, (i.e., palmitoleate and oleate) from their SFA precursors (i.e., palmitate and stearate) [55]. Several studies confirmed the role of SCD1 in tumorigenesis, as it modulates the FA composition of cancer cells. Indeed, its expression is increased in many types of cancer [56–58], where it protects cells from death, in particular from ferroptosis, an iron-mediated cell death mechanism that determines the peroxidation of membrane lipids, mainly PUFAs, constituting membrane phospholipids [59, 60]. An increased activity of SCD1 can lead to an unbalanced composition of membrane phospholipids with higher abundance of MUFAs, reluctant to lipid peroxidation and therefore resistant to ferroptosis [61].

FA synthesis enzymes are regulated at the transcriptional level by sterol regulatory element-binding protein (SREBP) transcription factors [62]. It has been noted that SREBP increases phospholipid, TG, and cholesterol synthesis to promote cancer cell survival and tumor growth [63, 64].

*β*-oxidation is an energetic process, generating acetyl-CoA entry into the TCA cycle, for the production of ATP in the mitochondria. Along with glucose and glutamine, FAs represent one of the main sources of energy [40]. Instead, FAs esterification leads to the synthesis of substrates useful for the generation of other complex lipids, such as phospholipids, sphingolipids and glycerolipids. These complex lipids can also act as lipid signalers that modify membrane structures and promote gene transcription for cell growth, proliferation and differentiation [46, 65].

One of the metabolic fates of FAs consists in their assembly into triglycerides (TG), which represent the main form of storage and FA transport within cells and in plasma [66]. The first step of TG synthesis consists in the esterification of the long-chain acyl-CoA into Glycerol-3-phosphate (G3P), by the action of mitochondrial and microsomal G3P acyltransferase (GPAT) enzymes. This reaction catalyzes the synthesis of lysophosphatidic acid (LPA) which is subsequently acylated to form phosphatidic acid (PA) by the acylglycerol-3-phosphate acyltransferases (AGPAT) present in the ER membrane. PA can be dephosphorylated by phosphatide phosphohydrolase (PAP, also known as Lipin) to form diacylglycerol (DG), precursors for TG synthesis; DG acyltransferase (DGAT), on the other hand, determines the final stage of TG biosynthesis, catalyzing DG acylation. The newly synthesized TG molecules are then directed by the ER lipid bilayer to form cytosolic lipid droplets or can be directly excreted from the cell (Fig. 1A) [67].

### FA pathway alterations and targeting in MM

Accumulation of TG-based lipid droplets occurs in malignant PCs, which, in the case of starvation, provides the long-term energy source available to support cellular needs [68]. Indeed, when energy is required, FA degradation is activated to produce ATP molecules for rapid tumor cell proliferation and growth [69]. Besides as energy reservoir, FAs are also important for their role in maintaining the integrity of the cell membrane, as well as signaling molecules [70].

Tumor cells exhibit elevated rate of lipogenesis, which is mostly supported by de novo FAs synthesis, to sustain FA catabolism through *β*-oxidation, as alternative pathway to support cancer cell proliferation and growth. Notably, CPT1 family members, including three enzymes, represent the rate-limiting enzymes of fatty acid oxidation (FAO), whose overexpression has been associated to carcinogenesis and chemoresistance [71]. In line with these notions, it has been demonstrated that etomoxir inhibits FAO by blocking CPT1, thus reducing the intracellular ATP levels and inducing cell-cycle arrest in G0/G1 phase in MM cells [72]. Moreover, the combination of etomoxir and orlistat, a FASN enzyme inhibitor, had an additive effect on MM cell viability inhibition, through the reduction of p21 protein levels and the phosphorylation of Rb. Notably, orlistat-mediated inhibition of de novo FA synthesis sensitized MM cells to bortezomib, reducing PI resistance through activation of apoptosis [72].

The pivotal role of ACC1 in FA synthesis makes it a promising therapeutic target for various metabolic diseases such as non-alcoholic fatty liver disease, obesity and diabetes. Many tumors have a high energy flow and a strong dependence on FA synthesis, making ACC a valuable therapeutic target also in neoplasias [53]. We recently demonstrated that a MIR17 host gene (MIR17HG)-derived long non-coding RNA, named lnc-17–92, acts as a chromatin scaffold for the functional interaction between the c-MYC onco-protein and WDR82, a regulatory component of the SET1 methyltransferase complex which catalyzes histone H3 Lys-4 (H3K4) methylation (mono-, di-, tri-) at the transcriptional start sites of active loci, thus promoting the expression of ACACA gene, encoding ACC1. Targeting MIR17HG pre-RNA with novel antisense molecules (ASOs) disrupted the transcriptional and functional activities of lnc-17–92, causing potent anti-tumor effects in preclinical models both in vitro and in vivo, which was associated with a decrease in de novo lipogenesis [73].

Under conditions of metabolic stress, such as hypoxia, glucose deprivation, and low serum, ACSS2 enzyme can promote malignant cell growth and survival in some solid tumors through the production of acetyl-CoA, which is further incorporated into metabolic pathways or acts as an epigenetic modulator in the activation of protein acetylation [74]. Interestingly, ACSS2 expression promoted myelomatous tumorigenesis through stabilization of IRF4, an oncogenic protein controlling a wide variety of genes that are key for cellular growth, survival and metabolic processes [75, 76]. In obese MM patients, the expression of ACSS2 was significantly higher than healthy individuals, and the increased ACSS2 level was positively correlated with high BMI values, thus strengthening the link between obesity and MM [76].

Reprogramming of lipid metabolism and changes in mitochondrial functions can also occur as adaptive survival mechanisms in MM cells after PIs treatment [77, 78]. Xu et al. demonstrated that PIs induce an abnormal lipid accumulation in MM cells, mainly observed in TGs content, through the activation of ATF4/SREBP pathway, conferring PIs resistance, as a compensatory mechanism to allow protein synthesis, avoiding ER stress and maintaining cell survival. On this basis, the authors explored the potential synergistic effects of lipid-modulating drugs in combination with PIs. The treatment with lovastatin, a well-known drug used for the treatment of hypercholesterolemia, in combination with PIs synergistically reduced MM cell viability, leading to a reduction of phospholipid-based metabolic flux and exacerbating the TG-based lipid content in malignant PCs, likely triggering a protective mechanism responsible for the adaptive survival of tumor cells. Based on the lovastatin-mediated increase in TG content, the administration of the chemically modified TG-lowering drug fenofibrate, a lipid-regulating fibric acid derivative used for the treatment of patients with hypertriglyceridemia, resulted in a strong synergistic anti-tumor effect, maximizing the killing of MM cells, when combined to lovastatin and PIs [79]. In parallel, Lipchick et al. reported that MM cells can reprogram their metabolism to develop PI resistance by inhibiting lipid synthesis, as demonstrated by the findings that levels of SREBP1, along with its downstream target fatty acid elongase 6 (ELOVL6), were lower in bortezomib-resistant than sensitive primary MM cells, reducing lipid synthesis and causing bortezomib resistance [80].

The abnormal FA metabolism in tumor cells is also linked to the altered activity of ACSL enzyme family [81]. A dual function of ACSL4 enzyme has been demonstrated in MM, with a tumor promoting function observed when highly expressed in malignant PCs, where its knock-down antagonizes cell proliferation, likely by inhibiting c-Myc and SREBPs transcription factor activity; on the other hand, high expression of ACSL4 is also mandatory for the activation of the ferroptosis pathway: as a consequence, ACSL4 knockdown induced ferroptosis resistance in MM cells, implicating ACSL4 as a predictive biomarker of ferroptosis sensitivity in MM [82].

Increasing evidence highlighted the role of PUFAs in determining cancer risk and progression [83]. An elevated expression of the cytosolic phospholipase A2*α* (cPLA2α), the rate-limiting enzyme involved in the production of arachidonic acid (AA), has been reported in both MGUS and MM patients. The authors demonstrated that the inhibition of cPLA2α reduced the viability of different MM cell lines, through activation of apoptotic pathway. Specifically, cPLA2α inhibitors, AVX420 and AVX002, downregulated the cellular content of PGE2, thus reducing the AA production as well as the expression of COX-2 and NF-κB, through the inhibition of PI3K/AKT signaling [84].

Recently, a role of fatty acid binding proteins (FABPs) in contributing to cancer development and progression has emerged [85–87]. FABPs facilitate the long-chain FAs intracellular trafficking and regulate FA synthesis and β-oxidation [86]. A recent study, carried out through an integrative analysis of MM and normal BM specimens in two published datasets, showed that FABP5 mRNA expression was significantly correlated with the infiltrations of immune cells, such as B cell naïve, macrophages M1, macrophages M2, neutrophils, activated NK cells and resting memory T cells, in the BMM of MM patients. This interplay acts as immunosuppressive mechanism during tumor progression, by which MM cells can regulate the function of immune cells, and is associated with unfavorable outcomes in patients, making FABP5 a candidate prognostic marker in MM [88]. Furthermore, Farrell et al. demonstrated that FABP inhibitors, BMS3094013 and SBFI-26, represent an effective strategy to target MM progression, both in vitro and in vivo, impacting on oncogenic c-Myc signaling pathway [89]. The inhibition of FABPs family, particularly FABP5, altered cell structure, inflammatory and metabolic pathways, clearly impacting on the mitochondrial membrane potential, oxygen consumption rates and FA oxidation (Fig. 1B) [90].

### Mevalonate

Cholesterol can enter cells via LDL cholesterol receptor-mediated uptake from the bloodstream or being synthesized de novo via the mevalonate biosynthetic (MVA) pathway, also called isoprenoid biosynthetic pathway (IBP), necessary for isoprenoid production, protein prenylation and production of coenzymes and other molecules [91]. This process occurs mainly in the liver, but also in other tissues such as the adrenal glands and the intestine. Its synthesis starts from acetyl-CoA, produced by the metabolism of carbohydrates and fats, and involves more than 20 enzymes distributed across the cytosol and the ER, leading to the formation of 3-hydroxy-3-methylglutaryl coenzyme A (HMGCoA) from three acetyl-CoA molecules, catalyzed by HMGCoA synthase (HMGCS) [92]; subsequently, 3-hydroxy-3-methylglutaryl-CoA reductase (HMGCR) converts HMG-CoA to mevalonate, which is phosphorylated by MVA kinase (MK) and converted into isopentenyl pyrophosphate (IPP).

Physiologic regulatory mechanisms maintain a balance in the synthesis and metabolism of cholesterol. One of the main regulatory pathways involves the HMGCR, which catalyses a key reaction in cholesterol synthesis. When cholesterol levels are high, this enzyme is inhibited by cellular signals that reduce its activity, thereby reducing the production of cholesterol.

IPP and dimethylamine pyrophosphate (DMAPP) serve as substrates for farnesyl diphosphate synthase (FDPS), to generate both geranyl pyrophosphate (GPP) and farnesyl pyrophosphate (FPP), which can be converted into squalene, a long-chain molecule that represents a key step in cholesterol synthesis; finally, by cyclization and side chain remodeling and after the action of squalene synthase and squalene epoxidase, squalene is converted into cholesterol [91].

Intermediates of the MVA pathway can undergo further enzymatic actions that catalyze the synthesis of molecules that participate in other biological mechanisms. For example, the synthesis of FPP and geranylgeranyl pyrophosphate (GGPP) is essential for protein prenylation, a post-translational modification required for the function of many signaling proteins, some of which belong to the Ras small GTPase superfamily (e.g., the Ras, Rab, and Rho), including over 150 proteins regulating many cellular functions, such as membrane trafficking, cell proliferation, survival and differentiation [93].

In addition to cholesterol synthesis, the mevalonate pathway is essential for the production of selenoproteins, including GPX4, a protein involved in cellular protection against ferroptosis [43]. The main regulators of cholesterol synthesis are sterol regulatory element binding proteins (SREBPs), which transcriptionally control the enzymes involved in the synthesis of sterols [61]. SREBP2 activates the transcription of enzymes of the mevalonate pathway, such as HMGCR, and regulates the uptake of cholesterol through the induction of low-density lipoprotein receptor (LDLR) expression [94]. On the other hand, LXRs control the reverse cholesterol transport pathway, through which the excess of cholesterol is returned to the liver for excretion as bile acids [95]. In conditions of low cholesterol levels, SREBP2 remains blocked in the ER, and oxysterols or desmosterol [96] bind and activate the LXR/RXR heterodimers, which in turn activate specific LXR target genes, such as ATP-binding cassette transporters A1 and G1 [97], ultimately promoting the elimination of the excess of cellular cholesterol (Fig. 1B) [98].

### Mevalonate pathway alterations and targeting in MM

Statins are well-known inhibitors of HMGCR as well as of the mevalonate pathway rate-limiting enzymes, which are crucial for cell growth and survival. Statins are used in the treatment of dyslipidaemia and coronary heart disease, although recently their application has emerged against various types of cancer, such as pancreatic ductal adenocarcinoma, prostate cancer, hepatocellular carcinoma, breast cancer stem cells, triple negative breast cancer, colorectal cancer, and many others [99]. In MM cell lines, HMGCR inhibitors induced apoptosis and activation of the unfolded protein response (UPR) pathway, inhibiting the production of FPP and GGPP isoprenoids through suppression of farnesylation and geranylgeranylation processes [100].

In addition, therapeutic combinations of various classes of statins (such as simvastatin, lovastatin, and fluvastatin) with the most widely used drugs in MM, such as lenalidomide, thalidomide, and bortezomib, displayed a synergistic reduction in cell viability and migration, by inducing over-expression of stress response genes and apoptosis. Moreover, treatment with statins was also effective in murine models of MM, as it reduced tumor burden and increased animal survival, either alone or in combination with bortezomib [101]. In line with these findings, in a cohort study of 4957 patients with MM, Sanfilippo et al. reported that statins improved survival of MM patients, with a 21% reduction in all-cause mortality and a 24% reduction in specific MM mortality [102].

Several studies demonstrated the beneficial effects of the therapeutic targeting of IBP signaling at the level of farnesyl diphosphate synthase (FDPS), which can be inhibited by bisphosphonates (BP) or nitrogen bisphosphonates (NPBs), the drugs most commonly used in the management of MM bone disease, acting by reducing bone resorption and increasing bone density through inhibition of osteoclast activity [103].

Hyperactivation of the RAS/RAF/MEK/ERK cascade associated with Ras mutations is constantly observed in MM patients, of which approximately 23% carry Ras mutations, in particular K-Ras mutations, which correlate with poor prognosis in MM. Such over-regulation is driven by MM-induced changes in the bone, resulting in the production of high levels of interleukins. The development of RAS inhibitors is an attractive area of investigation, although limited to the only approved inhibitor of G12C mutated K-Ras. An additional approach to modulate Ras expression is to inhibit Farnesyltransferase (FTase), which is fundamental in many biological aspects, including gene transcription regulation, intracellular signaling, protein–protein interactions and RAS membrane association. To date, Farnesyltransferase inhibitors (FTIs) have been assessed in clinical studies and demonstrated activity in breast cancer, myelodysplastic syndrome, and leukemias. Interestingly, the FTI called Tipifarnib (R115777) showed promising biological activity against myelodysplastic cells and led to significant clinical improvements of patients treated with doses below maximum tolerability [104]. In addition, the combination of Tipifarnib and bortezomib showed enhanced anti-MM activity respect to single agent treatment, as it enhanced the activation of the ER stress response, the induction of apoptosis as well as the reversal of cell-adhesion mediated drug resistance [105].

Additional therapeutic targets within the IBP pathway are geranylgeranyltransferase type I and II (GGTase I and II) enzymes, which are essential for Rho and Rab protein prenylation and geranylgeranylation, promoting their localization in the membrane and loading of GTP, ultimately activating signaling pathways driving actin polymerization. The development of GGTase I (GGTI-1) inhibitors proved difficult due to the strong similarity between the active site of GTase I and FTase causing off-target effects, thus making difficult the development of selective inhibitors. On the other hand, several inhibitors of GGTase II (GGTI-2) were developed and evaluated in preclinical studies. GGTase II inhibitors proved similar to statins treatment, in that they induced the accumulation of light intracellular chains and apoptosis mediated by UPR [101]. The first characterized GGTI-2 inhibitor was 3-PEHPC, an analog of biphosphonate known as risedronate, capable to inducing apoptosis and preventing Rab6 geranylgeranylation in MM cell lines, whose effect was unfortunately limited to preclinical studies due to the reduced potency (IC50 = 600 µM). Indeed, the development of a more potent analog of 3-PEHPC, carrying structural changes, yielded better anti-MM efficacy. Moreover, additional compounds were developed to optimize the inhibition of Rab activity, such as triazole inhibitors, N-oxide derivatives of 3-PEHPC, and benzimidazole carboxyphosphonates [106].

The geranylgeranylation of Rho and Rab proteins can also be targeted by geranylgeranyl diphosphate synthase (GGDPS) inhibitors (GGSI). GDPS catalyzes GGPP synthesis, which in turn is covalently bound to Rab proteins targeted by GGTase II. By inhibiting GGDPS in MM cells, the transport of monoclonal proteins (MP) in intracellular vesicles, which normally depends on the activity of Rab proteins, is impaired. Because of this deficit in Rab-mediated transport, monoclonal proteins are not properly directed to their cellular destinations; as a result, they accumulate within the RE, causing ER stress, activation of the UPR, and apoptosis [101].

One of the most powerful GGSI compound developed in recent years is VSW1198, a triazole isoprenoid composed of a mixture of homogeranyl and homoeryl isomers that synergistically interact to inhibit GGDPS. GGSI VSW1198 inhibited geranylgeranylation in MM cell lines and was highly selective to GGDPS [107]. In addition, a newly developed GGSI (CML-07–119) reduced serum M protein levels and interrupted Rap1a prenylation in transgenic mice [108]. Another GGSI (RAM2061) has been reported to slow tumor growth in a MM xenotransplantation model, dampening geranylgeranylation in vivo either alone or in combination with bortezomib [101].

### Sphingolipids

Sphingolipids play a structural role in the cytoplasmic membrane, but they are also implicated in the control of cell growth, proliferation and apoptosis [109]. These are amphipathic molecules, with ceramide representing the simplest one. Sphingolipid metabolism involves de novo synthesis in the endoplasmic reticulum (ER), where the enzyme serine palmitoyltransferase (SPT) catalyzes the coupling of palmitoyl coenzyme-A with the D-serine amino acid to synthesize 3-ketosphinganine [110]. Reduction of 3-ketosphinganine forms dihydrosphingosine, which is then typically N-acylated by one of six ceramide synthases (CerS1-CerS6) with 14–26 carbon saturated or monounsaturated fatty acids, to form dihydroceramides, subsequently dehydrogenated to ceramides by dihydroceramide desaturase [111].

Ceramides represent a crossroad for the production of other sphingolipids with different, even opposite activities [112]. The synthesis of sphingomyelin and a vast range of complex glycosphingolipids takes place from ceramide, which occurs mainly in the Golgi. Transport of ceramide from the ER to the Golgi occurs by ceramide transfer protein (CERT) for sphingomyelin, or by vesicular transport for glucosylceramide synthesis. Ceramides can also be phosphorylated in the Golgi apparatus by ceramide kinase (CERK) to form ceramide-1-phosphate (C1P); sphingomyelin and glycosphingolipids (GSL) are instead delivered to the plasma membrane by vesicular transport [111].

The activity of the ceramidase enzyme on ceramides allows the synthesis of sphingosine, which in turn can be phosphorylated by one of the two sphingosine kinases, SphK1 and SphK2, forming Sphingosine-1-phosphate (S1P), a metabolite of considerable interest because involved in many extra-metabolic cellular processes, such as inflammation, proliferation and angiogenesis [113]. S1P can have intracellular actions, can be exported to the extracellular environment through channels as the ATP-independent multi-transmembrane transporters Spinster homolog 2 (SPNS2), or can even be exocytosed via vesicles and/or lipid exosomes [114]. Once in the extracellular space, S1P binds to G protein-coupled S1P receptors, a family that is intertwined with other cellular signaling pathways, while intracellular S1P binds directly to intracellular protein targets (Fig. 1C) [115].

### Sphingolipid pathway alterations and targeting in MM

Sphingosine-1 phosphate (S1P) has been found involved in the interaction between tumor cells and the BMM [116].Intriguingly, while S1P represents a lipid capable of promoting tumorigenesis, ceramide represents a powerful anti-inflammatory and pro-apoptotic agent, hence the various enzymatic modifications that ceramide can undergo may lead to different and even opposite effects.

S1P is a bioactive lipid produced by several cell types present in the BM niche, including MM cells, polarized macrophages, osteoblasts and osteoclasts; it is involved in the processes of inflammation, proliferation and angiogenesis [114]. The binding of S1P to its membrane G protein-coupled receptors regulates cellular processes such as survival and proliferation through ERK1/2, PI3K, Rac and Rho activation.

Noteworthy, S1P has been reported to regulate hematopoietic progenitor cell lineage and plasma cell localization in the BM [117]. The work by Petrusca et al. highlights S1P as fundamental to send pro-tumor signals by increasing levels of the Growth Factor Independence 1 (GFI1) transcription factor, which consequently leads to increased growth and viability of MM cells, making MM cell resistant to bortezomib-induced cell death and also promoting osteoclastogenesis [112].

Due to S1P synthesis, sphingosine kinase (SphK) enzymes may represent key targets in reducing MM cell viability. Accordingly, K145, an inhibitor of sphingosine kinase 2 (SphK2), triggered MM cell death through UPR activation; moreover, the concomitant treatment of K145, a SphK2 inhibitor, together with bortezomib reactivated bortezomib sensitivity of drug-resistant MM cells [118]. An increased expression of SphK2 was found in MM cells as well as in MM patient PCs, where short hairpin RNA-mediated knock-down, or pharmacological inhibition of SphK2 by the selective inhibitor ABC294640, induced apoptosis via caspase-3 activation, overcoming the protective effect of BMSCs; additionally, ABC294640 enhanced c-Myc proteasome degradation [119].

During MM progression, there is a constant increase in angiogenesis in the BM niche, which is essential for MM growth, invasion and metastasis. The increased rate of angiogenesis represents the main difference between clinically active MM versus SMM, whereby an imbalance of pro- and anti-angiogenic factors determines the so-called angiogenic switch [120]. The SphK1-S1P-S1PR1 pathway has been found implicated in the regulation of angiogenesis: indeed, studies conducted in vivo demonstrated that mice with a general knockout of S1PR1 died from embryonic hemorrhage due to lack of vascular maturation [121].

Regarding the possible link of sphingolipids with drug resistance, it has been reported that sphingomyelin synthesis is associated with the onset of PI resistance in MM cells [78]. Accordingly, the SMS inhibitor D609 determined a greater cytotoxic effect in cells resistant to the bortezomib and carfilzomib compared to sensitive cells, suggesting a possible dependence of PI resistant cells on sphingomyelin [112].

The clinical confirmation of the close link between MM and sphingolipids comes from the increased risk of developing MM in patients with Gaucher disease, a sphingolipidosis that causes an accumulation of glucosylceramide due to an enzymatic deficiency of acid glucocerebrosidase [122]. Gaucher disease is likely associated with a high risk of monoclonal gammopathy due to the reduced presence of ceramide, whose activity triggers pro-apoptotic and anti-inflammatory effects also dampening chemoresistance [123].

Faict et al. also demonstrated dysregulation of sphingolipid metabolism due to an upregulation of acid sphingomyelinase (ASM) in primary MM cells as well as bortezomib-treated cell lines. Interestingly, the exosomes of these cells were also rich in ASM, suggesting the possibility of conferring the drug-resistant phenotype on other cells as well [124].

Some research groups have investigated the possible anti-angiogenic role of some classes of sphingolipids, including ceramide. Treatment of MM cells with C6-ceramide (ExoC6-cer), used to mimic the endogenous effects of ceramide, led to the inhibition of MM cell proliferation [125], as well as anti-angiogenic effects, likely through upregulating the exosomal levels of some tumor suppressive miRNAs (miR-29b, miR-202 and miR-15a/16), among which miR-29b, an established tumor suppressive miRNA in MM [126–129], accounted for the downregulation of AKT3, PI3K and VEGFA.

MM is associated with osteolytic bone lesions and skeletal complications [130]. Interactions between MM cells and BMSCs promote tumor growth, survival and bone destruction. Osteolytic lesions are localized to areas adjacent to tumor growth and are characterized by increased activity of osteoclastic bone resorption (activated by RANKL and IGF-I) and suppression of osteoblastogenesis [130, 131]. Osteoclasts are formed by fusion of mononuclear precursors and are responsible of the bone resorption. Lactosylceramide (LacCer), gangliosides GM2, and GM3 are the main GSL constituents of mature osteoclasts [132], while GM1 colocalizes with RANK, the RANKL receptor, in lipid rafts [133]. Exogenous GM3 and GM1 were able to restore osteoclast formation but less than LacCer [132], with GM3 acting as pro-osteoclastogenic factor synergistically enhancing the ability of RANKL and IGF-I to induce the maturation of osteoclasts. The increasing levels of GM3, often detected in MM patients, would be thus indicative of bone loss due to excessive osteoclast activity and failure of osteoblast activity. GSL synthesis inhibitors are also able to regulate osteoclastogenesis by interfering with RANK, c-Src and TRAF6 co-localization in the lipid rafts, ultimately interfering with the signaling cascade that activates the NF-kB pathway and the subsequent transcription of osteoclastogenic genes [134]. Inhibition of GSL synthesis using NB-DNJ, a selective GCS inhibitor, dramatically inhibited RANKL-induced osteoclastogenesis [135]. Elegant preclinical studies demonstrated that Eliglustat, an FDA-approved small GSL synthetic inhibitor used for the treatment of Gaucher disease type 1 in adults [136], was capable to reduce osteoclast-driven bone loss in MGUS and MM models, acting as autophagy inhibitor which prevents TRAF3 degradation through a GLS-dependent mechanism [137].

## Impact of lipid molecules on BMM fitness

The transfer of mitochondria among tumor cells and other cells of the microenvironment can indeed contribute to cancer-associated metabolic alterations. In MM, the uptake of BMSC-derived mitochondria, mechanistically mediated by tuneling nanotubes, has been associated to drug resistance due to increased ATP levels and reduced mitochondrial superoxide species [138, 139]. Within the BMM, MSCs are an essential precursor to BM adipocytes and osteoblasts. Increasing evidence indicates that the balance between the pool of MSCs and mature cells (adipocytes and osteoblasts) is often altered during aging and disease; moreover, MM-associated MSCs are distinct from healthy donor MSCs, and their gene expression profiles may be predictive of patient outcomes. Fairfield et al. demonstrated that MM cells can inhibit adipogenic differentiation while stimulating a senescence-associated secretory phenotype (SASP) prompting MM survival. Accordingly, the contact with MM cell lines, as well as MM-derived conditioned media, triggered a marked decrease in lipid accumulation in differentiating murine pre-adipocytes, which displayed gene expression changes in steroid biosynthesis, cell cycle and metabolism (oxidative phosphorylation and glycolysis) and a marked increase of typical MM-supportive genes, including IL-6 and CXCL12. Indirect exposure to MM-derived media, prior to differentiation induction, promoted a senescent-like phenotype in differentiating MSCs, and this trend was also confirmed in MM-associated MSCs compared to MSCs from normal donors [140].

FA metabolism in MM can be likely suggestive of a possible metabolic shift from aerobic glycolysis to FA oxidation, as previously reported in leukemia. Although MM cells heavily depend on glucose uptake, they lie in a niche surrounded by adipocytes [141], which occupy approximately the 70% of BM volume, and contain TGs that produce and release FAs, in turn taken up by MM cells [45, 141]. BM adipocytes (BMAds) have been shown to contribute to malignancies such as acute myelogenous leukemia, bone-metastatic breast cancer and MM, although the underlying mechanisms remain elusive [142]. After the initial discoveries indicating that BMAds support proliferation, migration and chemoresistance of MM cells [143, 144], it was reported that MM cells can in turn affect BMAds. Edwards group showed that, in the BM surrounding the tumor, BMAd number was decreased in mice with high MM burden [145], and that lipid content of BMAds was reduced after contact with MM cells. Furthermore, when co-cultured with MM cells, normal BMAds were reprogrammed and produced adipokines, such as adiponectin and TNF*α*, that activate osteoclastogenesis prompting MM bone disease [146].

Interestingly, Panaroni et al. demonstrated that BM aspirates from precursor states of MM, including MGUS and SMM, are more prone to adipogenic commitment compared with healthy donors. In vitro co-culture assays confirmed an adipocyte-induced promotion of MM cell proliferation in MGUS and SMM compared with NDMM. Using murine MM cell/BM adipocyte co-culture models, MM cells were found to induce lipolysis in adipocytes via activation of the ferroptosis pathway. Upregulation of fatty acid transporters 1 and 4 on MM cells mediated the uptake of secreted FFAs by adjacent MM cells. The effect of FFAs on MM cells was dose-dependent, with increased in proliferation at lower concentrations *versus* induction of ferroptosis-mediated lipotoxicity at higher concentrations. Intriguingly, exogenous administration of arachidonic acid, a very-long-chain FFA, in a murine plasmacytoma model triggered a reduction in tumor burden [69], strengthening the therapeutic potential of strategies modulating FAs in the BMM.

Immune dysfunction is present in MM, conferring tumor growth and resistance to therapy, but also determining a higher susceptibility of patients to infections and impaired cellular immunity, evidenced by a weak immune response to vaccinations [147, 148]. Overall, alterations in a wide variety of cells, including accessory and immune cells of the BM, as regulatory T cells, myeloid-derived suppressor cells [149, 150], Th17 cells [151], tumor-associated macrophages, MSCs and osteoclasts contribute to immune suppression and immune exhaustion found in MM [120]. Understanding metabolic alterations underlying T cell dysfunction in the BMM can be thus instrumental for the design of novel cancer immunotherapeutics. Intriguingly, decrease in BM CD8^+^ T-cell function in MM patients is common respect to healthy controls, and it is also consistently lower in BM samples than matched peripheral blood cells. These changes were accompanied by decreased mitochondrial mass and markedly elevated long-chain FA uptake. In vitro experiments confirmed that uptake of BM lipids suppressed CD8^+^ T function, which was impaired in autologous BM plasma, but rescued by lipid removal. RNA-sequencing revealed high expression of FATP1 in BM CD8^+^ T cells from MM patients, and FATP1 blockade rescued CD8^+^ T-cell function, thus representing a novel target for anti-MM immunotherapy. Notably, analysis of samples from treated patient cohorts demonstrated that CD8^+^ T cell metabolic dysfunction is relieved in treatment-responsive but not relapsed MM patients [152]. Increased CD36 expression in tumor-infiltrating CD8^+^ T cells was associated with tumor progression and poor survival in human and murine cancer models. Notably, CD36-depleted effector CD8^+^ T-cells exhibited increased cytotoxic cytokine production and enhanced tumor killing abilities; conversely, CD36-mediated uptake of FAs by tumor-infiltrating CD8^+^ T cells induced lipid peroxidation and ferroptosis, and led to reduced production of cytotoxic cytokines and impaired anti-MM activity [153].

Modulation of intracellular cholesterol levels can also trigger recognition and targeting of MM cells by NK cells, with potential implication in NK-based immunotherapy. As stated above, LXRs are nuclear receptors regulating intracellular cholesterol and lipid homeostasis. MM cells have been reported to respond to LXR activation through changes in the intracellular cholesterol content, associated with an enhanced expression of the NK cell-activating ligands, i.e. the major histocompatibility complex class I chain-related molecule A and B (MICA and MICB), two well characterized ligands of the NK group 2D receptor (NKG2D)/CD314 activating receptor expressed in cytotoxic lymphocytes, thus making MM cells more prone to NK-mediated recognition and killing. Mechanistically, LXR activation was found to regulate MICA at the transcriptional level, while MICB by inhibition of its lysosomal degradation [154].

## Circulating lipids as potential biomarkers in MM

Many studies have reported alterations in plasma lipid levels in many types of cancers, including MM, which can be regarded as novel potential prognostic, diagnostic and predictive circulating biomarkers.

The metabolic profile has proven useful to discriminate among healthy individuals, NDMM patients or those in complete remission [155]. By profiling the metabolome of BM supernatants and peripheral plasma of MM patients and healthy controls, Fei et al. identified potential diagnostic and prognostic biomarkers. In particular, a different metabolic pattern emerged between the two groups, highlighting the possible metabolic biomarkers associated with the risk of the disease, such as urea abundance, uric acid, serine and threonine in both plasma and BM of MM patients, likely associated with impaired kidney function during MM progression. In addition, FAs (linoleic acid, oleic and palmitic acid) and glycerol levels were significantly lower in MM patients than in healthy controls, while the levels of two TCA intermediates (succinate and malate) increased [156]. Additionally, patients with MM had higher saturated n-6 polyunsaturated FA and FA (PUFA) scores and lower monounsaturated, n-3 PUFA, and trans-FA scores than controls. The n-3/n-6 PUFA ratio was overall lower in patients with MM, and there was a distinct grouping of individual FA variants in MM patients [157].

FA levels in the erythrocytic membrane have been found altered in patients with MM compared to controls. Specifically, significant reductions in long-chain PUFA such as arachidonic acid and docosahexaenoic acid have been observed, representing potential diagnostic and/or predictive biomarker for MM [70].

Puchades-Carrasco et al. [158] used HNMR spectroscopy to characterize the different metabolic profiles of three patient groups consisting of 31 serum samples from healthy donors, 27 NDMM, and 23 patients after complete remission. NDMM patients had low levels of saturated, MUFA and PUFA, along with low levels of cholesterol, compared to the control group; conversely, patients in full remission showed an increase in cholesterol levels that matched the metabolic profiles of patients at the time of diagnosis.

Circulating lipids can be also used to improve the predictive accuracy of biomarkers for prognosis, likely helping the decision-making process and overall management of patients. In a prediction study of a cohort of 275 MM patients, a six parameters prognostic model, including ApoB, apoa1, LDL, HDL and TG, was capable of stratifying patients into high- and low-risk groups and predicting survival with greater accuracy and discriminatory ability than conventional (ISS and Durie Salmon) staging systems. Among 275 patients, 179 were treated with conventional drugs, while 96 underwent therapy with bortezomib. In both subgroups, patients in the low-risk group had longer overall survival; in addition, ApoB and the ApoB/apoa1 ratio were risk factors for MM patients, and survival times were extended when total cholesterol and HDL-C levels found elevated [159]. In a further study, 59 serum samples of MM patients before receiving treatment were analyzed to identify serum lipid biomarker candidates to predict the response to bortezomib. No significant difference was found between the responder and non-responder groups; while the levels of 10 lipid metabolites, 7 LPG (glycerophospholipids), of which two PCs represented phosphatidylcholine (PC:38:3/38:5), and five PE ethers of the phosphatidylethanolamine class (PE:36:4e/ 36:4p/ 38:4p/ 38:5p/ 38:7e), 1 SM of the sphingolipid class (SM 39:1 + H), and 2 ChE, cholesterol esters, (18:3 + NH4 and 20:3 + NH4), were progressively increased in non-responders, through minor/partial responders to good responders, thus emerging as potential predictive biomarkers of response to bortezomib therapy [160].

Other metabolites were found in different concentrations between the MGUS, NDMM, and RRMM groups such as free carnitine, acetylcarnitine, glutamate, asymmetric dimethylarginine (ADMA), and eight phosphatidylcholine species (PC), applying them as potential predictive biomarkers of disease [161].

Bone and lipid metabolic dysfunctions of MM patients have been associated with alterations in Vitamin D, a fat-soluble cell signaling molecule responsible for several functions within the cell, such as the maintenance of homeostasis and metabolism of calcium and phosphate and the regulation of bone metabolism. Bao et al. demonstrated that serum vitamin D is related to cholesterol, and that serum cholesterol and triglyceride levels in patients with NDMM were significantly lower in the group with a lower ratio, compared to the group with a higher ratio, of vitamin D to telopeptide carboxyterminal type I collagen (b-CTX), a known marker of osteoclast activity. This finding supports the notion that vitamin D deficiency is linked to dysfunction in bone and lipid metabolism, and can predict the severity of the disease [162].

## Conclusion and future perspectives

Lipid reprogramming is emerging among the metabolic hallmarks of cancer, and can be also exploited by myeloma PCs to meet the requirements for survival, proliferation, chemoresistance and immune evasion. It has now become clear that the BMM can contribute to the metabolic rewiring of the MM cells, causing changes in metabolite levels, increased/decreased activity of metabolic enzymes and metabolic shifts [35]. Such metabolic adaptations will promote a pro-tumoral environment stimulating MM growth and drug resistance.

Overall, lipid metabolic vulnerabilities of MM involve enhanced lipid de novo synthesis and/or exogenous lipid uptake, due to the overexpression of FASN, ACC1, HMGCR, and CD36 or FABP proteins. Targeting of sphingolipids, which regulate relevant survival pathways in MM PCs, have been also considered in the design of strategies aimed at reactivating the sensitivity of the malignant MM clone to standard-of-care treatments [112].

The major lipid therapeutic vulnerabilities found in MM, and discussed in this review, are summarized in Table 1.

**Table 1:** Drugs targeting lipid-related molecules in MM

Drug(s)	Target	Molecular and biological effects in preclinical MM models	References
Etomoxir	CPT1	Suppression of FAO through inhibition of long-chain FAs import into mitochondria Arrest of cell cycle in G0/G1 phase and reduction of intracellular ATP levels	[72]
Orlistat	FASN	Blocking of TG hydrolysis and FA absorption Sensitization of MM cells to bortezomib through activation of apoptosis	[72]
ASO targeting lnc-17–92	ACC1	Inhibition of lnc-17–92 with antagonistic effects on the c-MYC-ACC1 axis In vitro and in vivo anti-tumor effects in preclinical MM models	[73]
AVX420 AVX002	cPLA2α	Downregulation of PGE2 and arachidonic acid intracellular content Inhibition of NF-κB activity through the suppression of PI3K/AKT signaling Reduction of cell viability through apoptosis activation	[84]
BMS3094013 SBFI-26	FABP5	Alteration of cell structure, inflammatory and metabolic pathways Reduction of oxygen consumption rate and FAO	[90]
Statins	HMGCR	Inhibition of FPP and GGPP production through inhibition of farnesylation. and geranylgeranylation processes Activation of UPR pathway and induction of apoptosis	[100]
Bisphosphonates	FDPS	Immunomodulatory effects and direct anti-MM activity Reduction of bone resorption through osteoclastogenesis blockade	[103]
R115777	FTase	Inhibition of MM cell viability Activation of ER stress response through suppression of Ras expression	[104]
3-PEHPC	GGTase II	Accumulation of intracellular light chains and apoptosis mediated by UPR Suppression of MM cell proliferation and apoptosis induction by preventing Rab activation	[101, 106]
GGSI VSW1198 CML-07–119 RAM2061	GGDPS	In vitro and in vivo inhibition of tumor growth Downregulation of Rab-mediated proteins monoclonal traffic Reduction of serum M protein levels Activation of UPR and ER stress and induction of apoptosis	[101, 107, 108]
K145 ABC294640	SphK2	Reduction of intracellular S1P levels and increase in ceramide Reactivation of bortezomib sensitivity in drug-resistant MM cells Activation of apoptosis in MM cells via caspase-3 triggering Enhancement of c-MYC proteasomal degradation	[119]
D609	SMS	Reduction of intracellular SM synthesis Increase in ceramide intracellular levels Cytotoxic effects against PI-resistant MM cells	[112]
NB-DNJ	GSL	Suppression of RANKL-induced osteoclastogenesis and reduction of bone destruction	[135]
Eliglustat	GSL	Suppression of RANKL-induced osteoclastogenesis and reduction of bone destruction; synergistic effect with zoledronic acid	[137]

Overall, manipulation and/or pharmacological targeting of lipid pathways in tumor cells might cause multiple effects. As an example, therapeutic strategies aimed at the inhibition of key rate limiting enzymes in lipid biosynthesis can reduce lipid availability and energy supply, while over-stimulation of some lipid levels can trigger ER stress; in parallel, disruption of mitochondrial oxidative homeostasis can lead to mitochondrial stress, and blocking lipid-related signaling pathways can even promote ferroptosis, a programmed cell death caused by excessive accumulation of iron-dependent lipid peroxides from PUFA. Since several hematological malignancies seem sensitive to ferroptosis, it is emerging as a promising therapeutic strategy indeed deserving thorough investigation also in MM [39], especially on the basis of the wide epigenetic reprogramming that ferroptotic agents might trigger in MM cells [163].

Recent studies have also highlighted the multifaceted activities of cholesterol, regulating cell integrity and inflammatory responses. As such, manipulation of cholesterol homeostasis has been shown to affect leukemic and MM cell survival, and to restore immune responses [154, 164–167]. Because of the complex regulation of cholesterol metabolism and the multiple mechanisms by which cholesterol interferes with tumor and immune responses, targeting cholesterol metabolism for cancer treatment remains challenging. Preclinical studies have shown successful anti-tumor immune responses when using drugs that interfere with cholesterol homeostasis combined with immune checkpoint blockers [168], prompting the translation of this strategy into clinical practice to treat MM patients undergoing immune checkpoint blockade. Lipophilic statins have unfortunately a short half-life, suggesting that HMGCR activity should rapidly recover, leading to resynthesis of cholesterol and isoprenoids; moreover, diets containing geranylgeraniol may overcome the blockade of isoprenoid synthesis carried out by statins. All these factors, which may account for the failure of some prospective clinical trials [169], should be considered when designing statin-based studies [98].

In conclusion, understanding the oncogenic mechanisms of lipid metabolism, and targeting lipid metabolism reprogramming to identify new MM dependencies, has important scientific significance and holds the potential for clinical translation. It can be hypothesized that a single lipid pathway inhibition might not be effective in inhibiting MM development, activating escape metabolic pathways. Therefore, combined inhibition of multiple pathways, involved in lipid metabolism, glucose and/or amino acid metabolism, should be explored in the near future in the context of MM preclinical and clinical studies.

## Data Availability

Not applicable to this article, as no new data were created or analyzed in this review.
